# H7N9 virus infection triggers lethal cytokine storm by activating gasdermin E-mediated pyroptosis of lung alveolar epithelial cells

**DOI:** 10.1093/nsr/nwab137

**Published:** 2021-07-30

**Authors:** Xiaopeng Wan, Jiqing Li, Yupeng Wang, Xiaofei Yu, Xijun He, Jianzhong Shi, Guohua Deng, Xianying Zeng, Guobin Tian, Yanbing Li, Yongping Jiang, Yuntao Guan, Chengjun Li, Feng Shao, Hualan Chen

**Affiliations:** State Key Laboratory of Veterinary Biotechnology, Harbin Veterinary Research Institute, Chinese Academy of Agricultural Sciences, Harbin 150069, China; State Key Laboratory of Veterinary Biotechnology, Harbin Veterinary Research Institute, Chinese Academy of Agricultural Sciences, Harbin 150069, China; National Institute of Biological Sciences, Beijing 102206, China; State Key Laboratory of Veterinary Biotechnology, Harbin Veterinary Research Institute, Chinese Academy of Agricultural Sciences, Harbin 150069, China; State Key Laboratory of Veterinary Biotechnology, Harbin Veterinary Research Institute, Chinese Academy of Agricultural Sciences, Harbin 150069, China; State Key Laboratory of Veterinary Biotechnology, Harbin Veterinary Research Institute, Chinese Academy of Agricultural Sciences, Harbin 150069, China; State Key Laboratory of Veterinary Biotechnology, Harbin Veterinary Research Institute, Chinese Academy of Agricultural Sciences, Harbin 150069, China; State Key Laboratory of Veterinary Biotechnology, Harbin Veterinary Research Institute, Chinese Academy of Agricultural Sciences, Harbin 150069, China; State Key Laboratory of Veterinary Biotechnology, Harbin Veterinary Research Institute, Chinese Academy of Agricultural Sciences, Harbin 150069, China; State Key Laboratory of Veterinary Biotechnology, Harbin Veterinary Research Institute, Chinese Academy of Agricultural Sciences, Harbin 150069, China; State Key Laboratory of Veterinary Biotechnology, Harbin Veterinary Research Institute, Chinese Academy of Agricultural Sciences, Harbin 150069, China; State Key Laboratory of Veterinary Biotechnology, Harbin Veterinary Research Institute, Chinese Academy of Agricultural Sciences, Harbin 150069, China; State Key Laboratory of Veterinary Biotechnology, Harbin Veterinary Research Institute, Chinese Academy of Agricultural Sciences, Harbin 150069, China; National Institute of Biological Sciences, Beijing 102206, China; State Key Laboratory of Veterinary Biotechnology, Harbin Veterinary Research Institute, Chinese Academy of Agricultural Sciences, Harbin 150069, China

**Keywords:** H7N9 virus, cytokine storm, gasdermin E, pyroptosis

## Abstract

The H7N9 influenza virus emerged in China in 2013, causing more than 1560 human infections, 39% of which were fatal. A ‘cytokine storm’ in the lungs of H7N9 patients has been linked to a poor prognosis and death; however, the underlying mechanism that triggers the cytokine storm is unknown. Here, we found that efficient replication of the H7N9 virus in mouse lungs activates gasdermin E (GSDME)-mediated pyroptosis in alveolar epithelial cells, and that the released cytosolic contents then trigger a cytokine storm. Knockout of *Gsdme* switched the manner of death of A549 and human primary alveolar epithelial cells from pyroptosis to apoptosis upon H7N9 virus infection, and *Gsdme* knockout mice survived H7N9 virus lethal infection. Our findings reveal that GSDME activation is a key and unique mechanism for the pulmonary cytokine storm and lethal outcome of H7N9 virus infection and thus opens a new door for the development of antivirals against the H7N9 virus.

## INTRODUCTION

Influenza A viruses circulate widely in nature and continue to present a challenge to human health. While the H1N1 and H3N2 viruses are still actively circulating in humans around the world, other subtypes of avian influenza viruses have crossed the avian–human species barrier and infected humans [1–5]; of particular note, the H5N1 and H7N9 avian influenza viruses have caused severe disease and deaths in humans [6–11], prompting the fear that these avian influenza viruses may cause future human influenza pandemics with disastrous consequences. Understanding the pathogenesis of the infections caused by these viruses will be helpful for antiviral drug development.

The H7N9 viruses that emerged in China in 2013 have caused 1568 human infections, including 616 fatal cases [12]. Most H7N9 patients developed severe pneumonia and acute respiratory distress syndrome [1,6–8]. Analysis of bronchoalveolar lavage samples has shown that cytokine/chemokine levels in the lungs increase by 1000-fold relative to those in plasma, and that these increased cytokine/chemokine levels in the lungs correlate with a less favorable or fatal outcome [13]. However, how these increased cytokine/chemokine levels in the lungs (referred to as a ‘cytokine storm’ in this manuscript) are triggered remains largely unknown. In this study, we used an animal model to explore the underlying mechanism of the aberrant innate immune response induced by the H7N9 virus, and gained important insights into the pathogenesis of H7N9 infection in humans.

## RESULTS

### H7N9 virus A/Anhui/1/2013 causes cytokine storm in mice

Mice have been widely used as an animal model for evaluating the pathogenicity of influenza viruses. We selected two H7N9 viruses: (i) A/Anhui/1/2013 (H7N9) (AH/1), isolated from a fatal human case in 2013 [1], and (ii) A/pigeon/Shanghai/S1421/2013 (H7N9) (PG/S1421), isolated from a pigeon in 2013 [14], and compared their replication, virulence and the cytokine response in C57BL/6 (B6) mice. Groups of 32 6-week-old B6 mice were inoculated with a 10^5.5^ 50% egg infectious dose (EID_50_) of AH/1 or PG/S1421, and 16 6-week-old B6 mice were inoculated with phosphate-buffered saline (PBS) as a control. Five mice in each virus-inoculated group were euthanized on days 3 and 6 post-infection (p.i.), respectively, and their organs were collected for virus titration. Six mice in each virus-inoculated group were observed for body weight change and death for 2 weeks. Lung lavage samples from six mice in each virus-infected group and the PBS control group were collected on day 6 p.i. for chemokine and cytokine analysis. Lungs from 10 mice in each group were collected on day 6 p.i. to test the wet-to-dry ratio (*n* = 5) and to quantify immune cell infiltration (*n* = 5).

**Figure 1.**
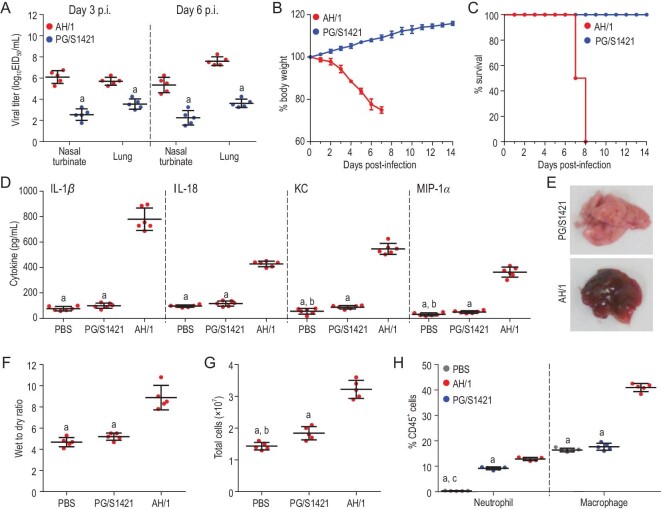
Replication and virulence of different H7N9 viruses in C57BL/6 (B6) mice. Groups of 32 B6 mice were inoculated intranasally with 10^5.5^ EID_50_ of PG/S1421 or AH/1, and 16 mice were inoculated with PBS as a control. Five mice in each virus-inoculated group were euthanized on days 3 and 6 post-infection (p.i.), respectively, and (A) their organs were collected for virus titration. Six mice in each virus-inoculated group were observed for (B) body weight change and (C) death for 2 weeks. (D) Lung lavage samples from six mice in each virus-infected group and the PBS control group were collected for analysis of chemokines and cytokines on day 6 p.i.; (E) lungs of the virus-inoculated mice or PBS-inoculated control mice were collected on day 6 p.i. to test (F) the wet-to-dry ratio (*n* = 5), (G) total cell and (H) infiltration of immune cell quantification (*n* = 5). Data in panels A–D, F and G are means ± standard deviation. Statistical analysis was performed by using the Student's t-test. a, *p* < 0.01 compared with the values of AH/1-inoculated mice; b, *p* < 0.05 compared with the values of PG/S1421-inoculated mice; c, *p* < 0.01 compared with the values of PG/S1421-inoculated mice.

Both viruses were detected in the nasal turbinates and lungs of the mice, but the titers of AH/1 were significantly higher than those of PG/S1421 (Fig. 1A). The mice in the AH/1-inoculated group experienced severe body weight loss and died within 8 days p.i., whereas the PG/S1421-inoculated mice gained weight and survived during the observation period (Fig. 1B and C). The levels of IL-1α, IL-1β, IL-6, IL-10, IL-12, IL-18, G-CSF, KC, MCP-1 and MIP-1α in the lungs of the AH/1-inoculated group were significantly higher than those in the lungs of the PG/S1421- and PBS-inoculated groups (Fig. 1D, Supplementary Fig. S1); the levels of only five cytokines and chemokines (KC, IL-6, G-CSF, MCP-1 and MIP-1α) in the lungs of the PG/S1421-inoculated group were significantly higher than those in the PBS-inoculated group (Fig. 1D, Supplementary Fig. S1). The lungs of the PG/S1421-inoculated mice appeared normal, whereas those of the AH/1-inoculated mice showed complete consolidation with edema and a red to dark-red appearance (Fig. 1E). The wet-to-dry ratio and total cell count results indicated that the lungs of the AH/1-inoculated mice experienced significant inflammatory exudates and massive immune cell infiltration (Fig. 1F and G); the numbers of both neutrophils and macrophages in the lungs of the AH/1-inoculated mice were significantly higher than those in the lungs of the PG/S1421-inoculated mice (Fig. 1H). These results indicate that the AH/1 virus, but not the PG/S1421 virus, causes a severe cytokine storm and is lethal in mice.

### AH/1 virus infection triggers GSDME-mediated pyroptosis in the lungs of mice

IL-1β and IL-18 are restricted to the cytosol because they lack a conventional secretion signal, and are only released when the cell membrane has been punctured or ruptured, for example, during pyroptosis [15–17]. Membrane rupture in pyroptosis results from activation of the gasdermin family of membrane pore-forming proteins, including gasdermin D (GSDMD), the substrate of caspase-1 and -11 in the inflammasome pathways [18–21]. Of note, the levels of IL-1β and IL-18 in the lung lavage samples from the AH/1-inoculated mice were significantly higher than those in the lungs of the PG/S1421- and PBS-inoculated mice, suggesting that cell leakage may have occurred in the lungs of the AH/1-inoculated mice. Cell leakage occurs when the integrity of the cell membrane is damaged. Recent studies indicate that certain cells express gasdermin E (GSDME, also called deafness autosomal dominant 5), which is specifically cleaved by activated caspase-3, and that the resulting N terminal of GSDME (Gsdme-N) then forms pores in the cell membrane and switches apoptosis to pyroptosis in response to caspase-3 triggers, such as chemotherapeutic drugs, tumor necrosis factor and viral infection [22,23]. The cytosolic contents released from the punctured or ruptured cells subsequently trigger an intense inflammatory response [24,25]. We therefore investigated whether the cytokine storm in the lungs of mice infected with the H7N9 virus resulted from GSDME-mediated pyroptosis.

**Figure 2.**
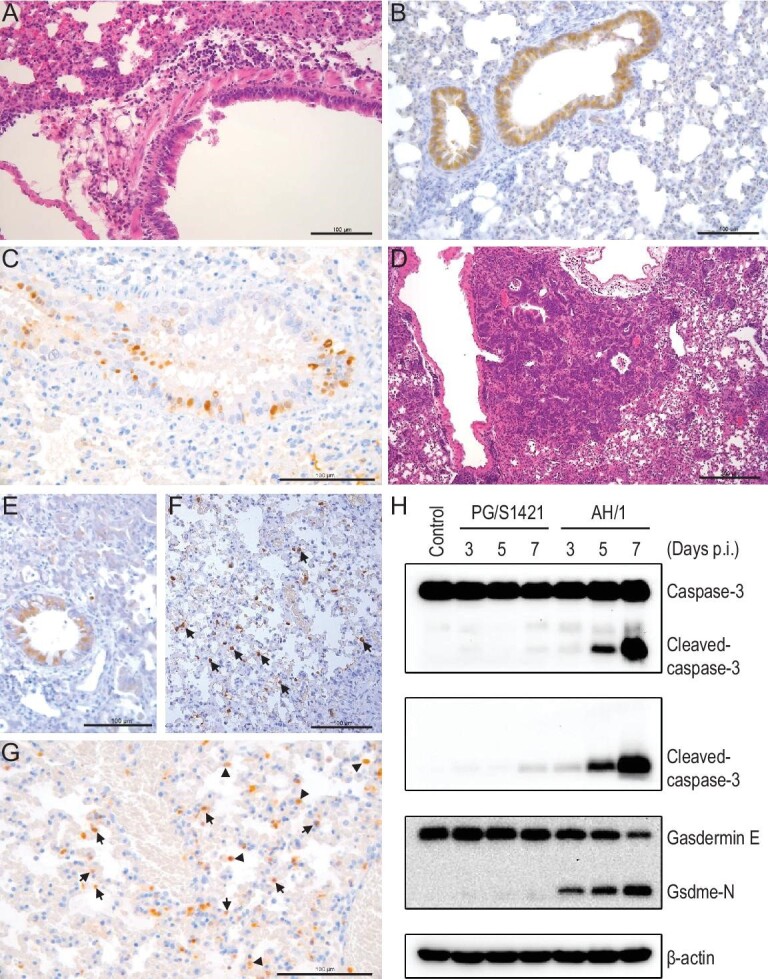
Caspase-3 and gasdermin E (GSDME) activation in the lungs of mice infected with different H7N9 viruses. Groups of 12 6-week-old B6 mice were inoculated with 10^5.5^ EID_50_ of (A–C) PG/S1421 or (D–G) AH/1. Three mice in each group were euthanized on day 6 p.i. and their lungs were collected for (A and D) pathologic study and immunohistochemical (IHC) study to detect (B, E and F) the viral antigen and (C and G) activated caspase-3. Three mice in each virus-inoculated group were euthanized on days 3, 5 and 7 p.i., respectively, and their lungs were collected for the detection of (H) caspase-3 and GSDME by use of SDS-PAGE and Western blotting. Scale bars in A–C and E–G = 100 μm; scale bar in D = 200 μm. Arrows and arrowheads on F and G indicate alveolar epithelial cells and macrophages, respectively.

To investigate caspase-3 activation and GSDME cleavage in mice inoculated with H7N9 viruses, groups of 12 6-week-old B6 mice were inoculated with 10^5.5^ EID_50_ of AH/1 or PG/S1421 virus. Three mice in each group were euthanized on day 6 p.i. and their lungs were collected for pathologic study and immunohistochemical (IHC) study to detect the viral antigen and activated caspase-3. Three mice in each group were euthanized on days 3, 5 and 7 p.i., respectively, and their lungs were collected for the detection of caspase-3 and GSDME by use of sodium dodecyl sulfate-polyacrylamide gel electrophoresis (SDS-PAGE) and Western blotting. As shown in Fig. 2, in the lungs of the PG/S1421-inoculated mice, mild-moderate peribronchiolar inflammatory infiltration with thickened alveolar walls was observed (Fig. 2A); viral antigen-positive signals and cleaved-caspase-3 staining were mainly detected in the epithelial cells of the bronchus (Fig. 2B and C). In the lungs of AH/1-inoculated mice, massive bronchiolization of the alveoli, intra-alveolar aggregates of macrophages and neutrophils, and perivascular edema and inflammatory infiltration were observed (Fig. 2D). Viral antigen-positive signals and cleaved-caspase-3 staining were not only present in the epithelial cells of the bronchus, but were also diffusely present in the alveoli and inter-alveolar septal walls (Fig. 2E–G). The protein levels of activated caspase-3 (cleaved-caspase-3) and the Gsdme-N cleavage product in the lung samples from AH/1-inoculated mice gradually increased over the days of infection, but this pattern was not observed in the samples from the PG/S1421-inoculated mice (Fig. 2H).

**Figure 3.**
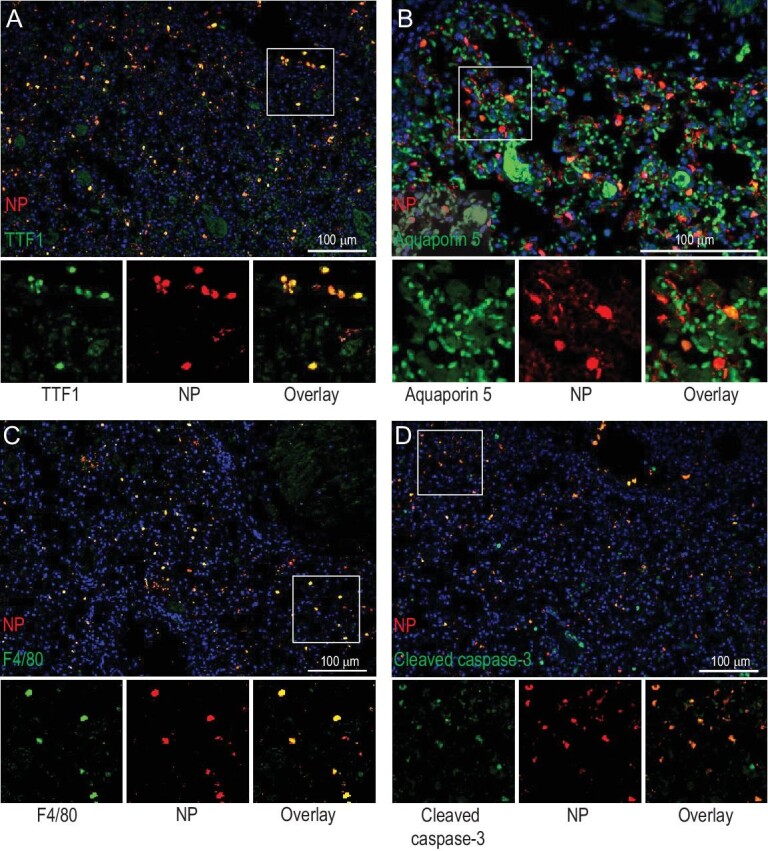
Identification of cell types infected by AH/1 virus in the lungs of mice. Dual-staining was performed on the lung sections of AH/1-infected mice to identify (A) the type II alveolar epithelial cells, (B) type I alveolar epithelial cells, (C) macrophages and (D) positive cleaved-caspase-3 staining cells that were viral antigen-positive. Scale bars: 100 μm.

Dual-staining analysis to identify the cell types that were infected by the AH/1 virus revealed that most of the viral antigen-positive cells were type II alveolar epithelial cells (Fig. 3A), although a small number of type I alveolar epithelial cells (Fig. 3B) and macrophages were also viral-antigen-positive (Fig. 3C). Most of the cleaved-caspase-3 staining cells were also viral-antigen-positive (Fig. 3D).

### Alveolar epithelial cells undergo GSDME-mediated pyroptosis during the AH/1 virus infection

Since the activated caspase-3 was detected in macrophages and alveolar epithelial cells in the lungs of the AH/1-inoculated mice, we performed a series of *in vitro* tests to identify the cells that underwent pyroptosis during the AH/1 virus infection.

**Figure 4.**
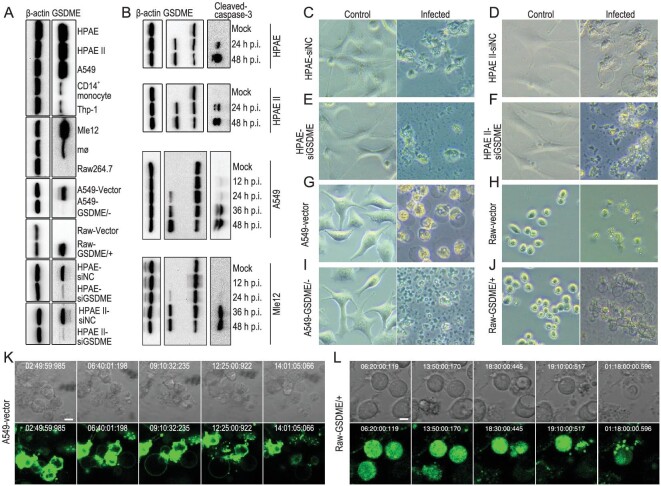
GSDME-mediated pyroptosis of different cells after H7N9 virus infection. (A) GSDME expression levels of different cells. (B) Activation of caspase-3 and Gsdme-N in different alveolar epithelial cells after AH/1 infection. (C, D, G and K) Different alveolar epithelial cells and (J and L) Raw-GSDME/+ cells undergo pyroptosis upon AH/1 infection, and their manner of death is correlated with the abundance of cellular GSDME, as evidenced by (E, F and I) GSDME-deficient alveolar epithelial cells and (H) Raw-vector cells undergoing apoptosis upon AH/1 infection. Static bright field cell images in panels C to J were obtained using the Zeiss primovert microscope and processed using zen blue software. The time-lapse phase-contrast and fluorescent images of cells in K and L were taken at the indicated timepoints after viral infection by using a PerkinElmer UltraVIEW spinning disk confocal microscope and processed by using the Volocity software. Scale bar in K and L: 5 μm.

We first compared the GSDME expression level in different primary cells and cell lines, including five kinds of human-origin cells [human primary alveolar epithelial (HPAE) cells, human primary type II alveolar epithelial (HPAE II) cells, and CD14^+^ monocytes, human alveolar epithelial cells (cell line) (A549), and macrophages (cell line) (Thp-1)] and three kinds of mouse-origin cells [a mouse alveolar epithelial cell line (Mle12), mouse primary macrophages (mø) and mouse leukemic monocyte macrophages (Raw264.7)]. We also generated and tested GSDME-knockout/knockdown and GSDME-overexpressing cell lines, including GSDME-deficient A549 cells (A549-GSDME/−), GSDME-overexpressing Raw264.7 cells (Raw-GSDME/+) and GSDME knockdown HPAE and HPAE II cells (HPAE-siGSDME and HPAE II-siGSDME) (Fig. 4A). We found that the GSDME expression in the HPAE cells, HPAE II cells and A549 cells was notably higher than in the CD14^+^ monocytes and Thp-1 cells, and that the GSDME expression in the Mle12 cells was notably higher than in the mø and Raw264.7 cells (Fig. 4A). GSDME expression was not detectable in the A549-GSDME/− cells and was detected at a very low level in the HPAE-siGSDME and HPAE-siGSDME cells, whereas it was highly expressed in the Raw-GSDME/+ cells (Fig. 4A). These results indicate that GSDME expression is more abundant in alveolar epithelial cells than in macrophages. Of note, GSDME expression in alveolar epithelial cells in normal human lung sections was also confirmed by IHC analysis (Supplementary Fig. S2). We further confirmed that the protein levels of the cleaved caspase-3 and the Gsdme-N in the AH/1-inoculated alveolar epithelial cells gradually increased over the time of infection (Fig. 4B).

We next investigated the morphological changes of different types of cells after AH/1 infection. We found that the four types of alveolar epithelial cells and the GSDME-overexpressing macrophage Raw-GSDME/+ cells swelled and blew bubbles from the plasma membrane (Fig. 4C, D, G, J, K and L; Supplementary Fig. S3A), showing typical pyroptotic features that have been described previously [18,26]. In contrast, the GSDME knockout and knockdown alveolar epithelial cells and the four types of macrophages shrank, formed small fragments and died mainly by apoptosis (Fig. 4E, F, H and I; Supplementary Fig. S3B–D). These results indicate that the morphological changes of these cells were directly related to GSDME expression.

**Figure 5.**
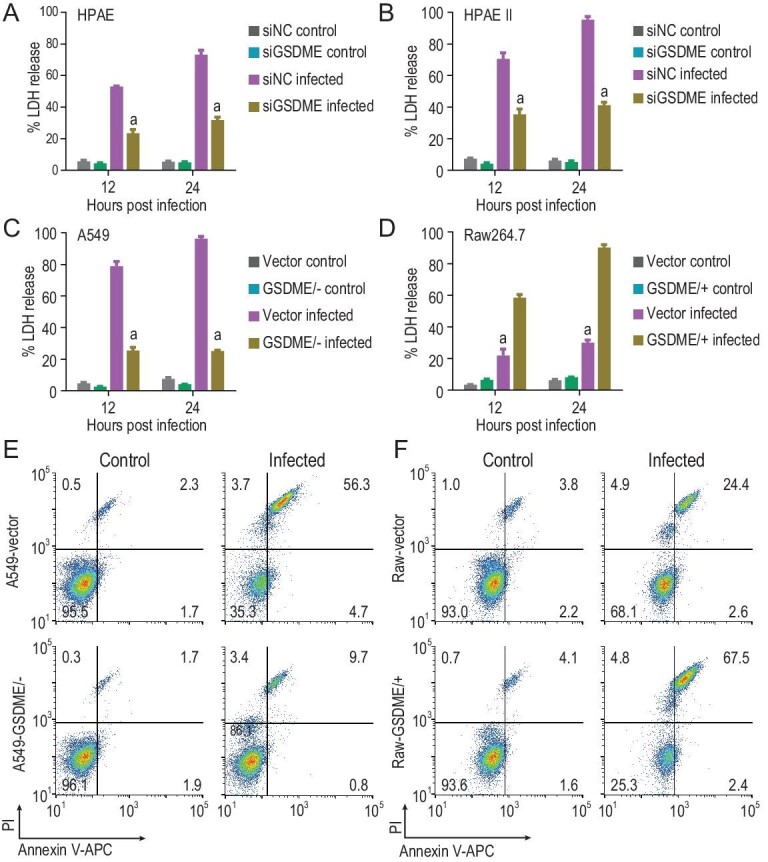
Lactate dehydrogenase (LDH) release and propidium iodide (PI) permeant of different cells after AH/1 infection. LDH release of different types of (A–C) alveolar epithelial cells and (D) macrophages were measured at the indicated timepoints after the AH/1 infection. PI permeant in different (E) A549 cells and (F) Raw246.7 cells were measured 24 h after the AH/1 infection. a, *p* < 0.01 compared with the values of their virus-infected control cell counterparts. All data are representative of three independent experiments.

When cell membranes are damaged, lactate dehydrogenase (LDH), a soluble enzyme found inside every living cell, is released into the surrounding extracellular space; therefore, the presence of LDH in culture medium is also used as a pyroptosis marker [27]. LDH-release from the AH/1-infected alveolar epithelial cells, including A549, HPAE and HPAE II cells, was significantly higher than that from their GSDME knockout or knockdown counterparts (Fig. 5A–C), and LDH-release from the AH/1-infected Raw264.7 cells was significantly lower than that from the AH/1-infected Raw-GSDME/+ cells (Fig. 5D).

Propidium iodide (PI) is a popular red-fluorescent nuclear and chromosome counterstain. Because PI is not permeant to live cells, it is commonly used to detect pyroptotic cells in a population. We found that, 24 h after AH/1 infection, the PI positive-stained populations of A549-vector control cells and A549-GSDME/− cells were 56.3% and 9.7%, respectively (Fig. 5E), and that the PI positive-stained populations of Raw-vector control cells and Raw-GSDME/+ cells were 26.4% and 67.5%, respectively (Fig. 5F).

We further evaluated the replication of the H7N9 virus in A549-vector, A549-GSDME/−, Raw-vector and Raw-GSDME/+ cells, and found that at 48 h after infection with AH/1 at a multiplicity of infection (MOI) of 0.5, the viral titer in the A549-vector cells was significantly higher than that in the A549-GSDME/− cells, and the viral titer in Raw-GSDME/+ cells was significantly higher than that in the Raw-vector cells, despite no significant difference at earlier timepoints (Supplementary Fig. S4).

These *in vitro* findings indicate that AH/1 virus infection induces pyroptosis in alveolar epithelial cells but apoptosis in macrophages, and that the manner of death largely depends on the level of cellular GSDME.

### *Gsdme* knockout protects mice from an H7N9 virus lethal infection

**Figure 6.**
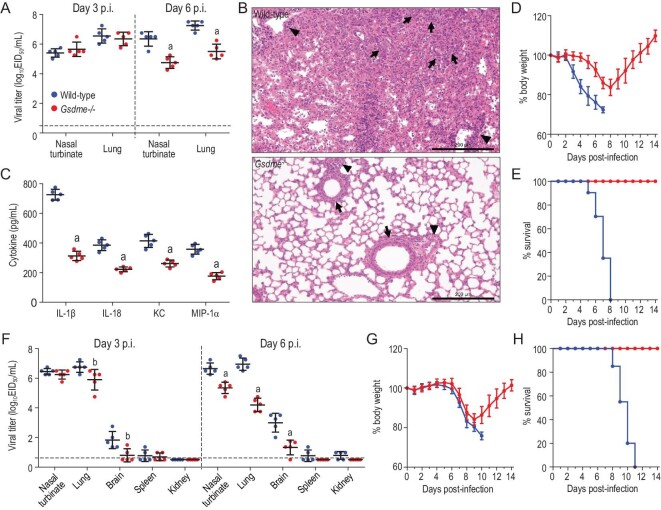
Replication and virulence of H7N9 viruses in wild-type B6 and *Gsdme*^–/–^ mice. Groups of 28 wild-type B6 mice and *Gsdme* knockout (*Gsdme*^–/–^) mice were intranasally inoculated with 10 MLD_50_ of AH/1 virus. (A) Five mice in each group were euthanized on days 3 and 6 p.i., respectively, and their nasal turbinates and lungs were collected for virus titration in eggs. (B) The lungs of three mice in each group that were euthanized on day 6 p.i. were collected for pathologic study; arrows indicate alveolar bronchiolization, arrowheads indicate perivasculitis. (C) Lung lavage samples from five mice from each group were collected for chemokine and cytokine analysis. The remaining 10 mice in each group were used to evaluate lethality and their (D) body weight change and (E) death were observed for 2 weeks. We repeated the lethality study one more time with groups of 10 mice, and the combined data are shown in D and E. (F–H) The replication and virulence studies in wild-type B6 mice and *Gsdme^–^^/^^–^* mice were also repeated with another H7N9 virus (CK/SD008-PB2/627K). Data in panels A, C, D, F and G are means ± standard deviation. Statistical analysis was performed by using the Student's t-test. a, *p* < 0.01 compared with the values of wild-type mice; b, *p* < 0.05 compared with the values of wild-type mice. Scale bar in B: 200 μm.

To further investigate the role of GSDME in the pathogenesis of H7N9 virus infection *in vivo*, groups of 28 *Gsdme* knockout (*Gsdme*^–/–^) mice [22] and their littermate wild-type B6 mice were intranasally inoculated with 10^5.5^ EID_50_ of AH/1 virus. Five mice in each group were euthanized on days 3 and 6 p.i., respectively, and their nasal turbinates and lungs were collected for virus titration in eggs. Eight mice in each group were euthanized on day 6 p.i.; the lungs of three of these mice were collected for pathologic study, and lung lavage samples from five of the mice were collected for chemokine and cytokine analysis. The remaining 10 mice in each group were observed for body weight change and death for 2 weeks. The viral titers in the nasal turbinates and lungs of the *Gsdme*^–/–^mice were similar to those of the wild-type mice on day 3 p.i., but were significantly lower on day 6 p.i. (Fig. 6A). Pathologic studies showed that the alveolar septal walls were thickened due to increased numbers of lymphocytes, macrophages and neutrophils in the lungs of the wild-type mice, whereas only mild lymphoplasmacytic peribronchiolitis and perivasculitis were observed in the lungs of the *Gsdme^–/–^* mice (Fig. 6B). The levels of IL-1α, IL-1β, IL-6, IL-10, IL-12, IL-18, G-CSF, KC, MCP-1 and MIP-1α in the lungs of the wild-type mice were significantly higher than those in the lungs of the *Gsdme*^–/–^ mice (Fig. 6C, Supplementary Fig. S5). The wild-type mice lost nearly 30% of their body weight and died within 8 days p.i., whereas the *Gsdme^–^^/^^–^* mice lost 18% of their body weight by day 8 p.i., but gradually recovered and all of them survived the 2-week observation period (Fig. 6D and E). We repeated the lethality test with 10 mice in each group, and the combined data are shown in Fig. 6D and E. These results indicate that GSDME-mediated pyroptosis plays a key role in the cytokine storm and lethal outcome of AH/1 virus infection in mice.

We previously reported that some H7N9 viruses isolated from poultry in 2017 obtained an insertion of four amino acids in their hemagglutinin (HA) cleavage site and were lethal in chickens [28]. Studies showed that these viruses readily obtained the 627K mutation in their PB2 upon replication in ferrets or humans, causing them to become highly lethal in mice and ferrets and to be transmitted efficiently in ferrets by respiratory droplet [28,29]. To further confirm the effect of GSDME on the lethality of H7N9 virus infection, groups of 20 *Gsdme^–^^/^^–^* mice and their littermate wild-type B6 mice were inoculated with a 10 50% mouse lethal dose (MLD_50_) (10^2.8^ EID_50_) of the highly pathogenic H7N9 virus CK/SD008-PB2/627K. Five mice in each group were euthanized on days 3 and 6 p.i., respectively, and their organs were collected for virus titration in eggs. On day 3 p.i., the CK/SD008-PB2/627K virus was detected in the nasal turbinates, lungs, brains and spleens of both wild-type mice and *Gsdme^–^^/^^–^* mice, but the viral titers in the lungs and brains of the *Gsdme^–^^/^^–^* mice were significantly lower than those in the wild-type mice (Fig. 6F); on day 6 p.i., the virus was detected in the nasal turbinate, lungs, brains, spleens and kidneys of the wild-type mice, and in the nasal turbinate, lungs and brains of the *Gsdme^–^^/^^–^* mice, but the viral titers in the *Gsdme^–^^/^^–^* mice were significantly lower than those in the wild-type mice (Fig. 6F). Ten mice from each group were tested for viral lethality and were observed for 2 weeks. The wild-type mice started to lose weight on day 6 p.i. and all died within 11 days p.i., whereas the *Gsdme^–^^/^^–^* mice experienced weight loss between day 6 p.i. and day 9 p.i., but then gradually regained weight and all of them survived the 2-week observation period. We repeated the lethality test with 10 mice in each group, and the combined data are shown in Fig. 6G and H). These results suggest that GSDME-mediated pyroptosis is a common mechanism for the lethal outcome of mice infected with different H7N9 viruses.

## DISCUSSION

In summary, we explored the underlying mechanism of the cytokine storm and lethal infection caused by H7N9 viruses, and found that mammalian-adapted H7N9 viruses can activate and switch caspase-3-mediated apoptosis to pyroptosis in GSDME-abundant alveolar epithelial cells. The GSDME-mediated pyroptosis in alveolar epithelial cells and cytosolic contents released from the ruptured alveolar epithelial cells triggered a cytokine storm together and contributed to the fatal outcomes in mice infected with the H7N9 virus. Recent studies have shown that a low level of tumor cell pyroptosis can cause a robust immune response sufficient for immune clearance of tumors in mice [30]. Indeed, activation of the caspase-3-GSDME axis by granzyme B [31] or GSDMB by granzyme A [32] from cytotoxic lymphocytes serves as the effector mechanism in antitumor immunity. Further, it is possible that activation of GSDME in H7N9-infected cells by cytotoxic lymphocytes may also contribute to the cytokine storm *in vivo* in addition to virus-induced direct activation of GSDME in lung epithelial cells.

The ability of the H7N9 virus to activate GSDME-associated pyroptosis in lung epithelium varies among strains. Two strains (AH/1 and CK/SD008-PB2/627K) killed mice by causing GSDME-induced pyroptosis in lung epithelium, whereas PG/S1421 did not activate this mechanism in the lungs of mice and was avirulent in these animals. A previous study by Kong *et al.* demonstrated that the single amino acid mutation of K627E in PB2 dramatically decreased the replication of the AH/1 virus in A549 cells [33]. PG/S1421 and AH/1 are similar in their genome, differing by only a single amino acid at position 627 in their PB2: PG/S1421 has 627E and AH/1 has 627K [14,33], suggesting two possibilities for how GSDME-mediated pyroptosis is activated by the virus. The first possibility is that PB2 with 627K directly plays a role in activation of GSDME-mediated pyroptosis. The second possibility is that the 627E to 627K mutation in PB2 increases the polymerase activity that promotes the replication of the H7N9 virus, and high viral loading in the lung triggers the activation. The fact that A549 cells infected with 100 MOI of PG/S1421 underwent a similar degree of pyroptosis as cells infected with 1 MOI of AH/1 (Supplementary Fig. S6) supports the second scenario.

Moreover, the H5N1 virus A/bar-headed goose/Qinghai/3/2005 (BHG/3), which also bears 627K in its PB2 and is highly lethal in mice [34], induced a similar degree of pyroptosis in A549 cells and A549-GSDME/− cells (Supplementary Fig. S7), and caused similar death patterns in GSDME-knockout mice and wild-type mice (Supplementary Fig. S8), demonstrating that the death in mice caused by H5N1 virus BHG/3 is not associated with GSDME-mediated pyroptosis and that PB2 with 627K does not play a direct role in the activation of GSDME-mediated pyroptosis. These results also suggest that different subtypes of influenza virus cause fatal infections via different mechanisms, which is also supported by the previous observation that both H1N1 and H5N1 viruses can induce autophagy in A549 cells, but only H5N1 virus infection can cause autophagic death in A549 cells [35].

GSDME activation is a key factor in the death of the host after H7N9 infection, suggesting that any strategy designed to prevent the activation of GSDME may alleviate the disease and improve the outcome of H7N9 virus infection. Our findings thus open a new door for the development of antiviral drugs against H7N9 virus infection.

## MATERIALS AND METHODS

Detailed descriptions of materials and methods are available as supplementary data at NSR online.

## DATA AVAILABILITY

All data are available in the manuscript and the supplementary materials.

## Supplementary Material

nwab137_Supplemental_Files

## References

[1] Gao R , Cao B, Hu Y et al. Human infection with a novel avian-origin influenza A (H7N9) virus. N Engl J Med 2013; 368: 1888–97. 10.1056/NEJMoa1304459 23577628

[2] Wong SSY , Yuen K. Avian influenza virus infections in humans. Chest 2006; 129: 156–68. 10.1378/chest.129.1.156 16424427 PMC7094746

[3] Chen H , Yuan H, Gao R et al. Clinical and epidemiological characteristics of a fatal case of avian influenza A H10N8 virus infection: a descriptive study. Lancet 2014; 383: 714–21. 10.1016/S0140-6736(14)60111-2 24507376

[4] Song W , Qin K. Human-infecting influenza A (H9N2) virus: a forgotten potential pandemic strain? Zoon Publ Health 2020; 67: 203–12. 10.1111/zph.12685 31930694

[5] Fouchier RA , Schneeberger PM, Rozendaal FW et al. Avian influenza A virus (H7N7) associated with human conjunctivitis and a fatal case of acute respiratory distress syndrome. Proc Natl Acad Sci USA 2004; 101:1356–61. 10.1073/pnas.0308352100 14745020 PMC337057

[6] Zhou J , Wang D, Gao R et al. Biological features of novel avian influenza A (H7N9) virus. Nature 2013; 499: 500–3. 10.1038/nature12379 23823727

[7] Yu L , Wang Z, Chen Y et al. Clinical, virological, and histopathological manifestations of fatal human infections by avian influenza A(H7N9) virus. Clin Infect Dis 2013; 57: 1449–57. 10.1093/cid/cit541 23943822

[8] Chen Y , Liang W, Yang S et al. Human infections with the emerging avian influenza A H7N9 virus from wet market poultry: clinical analysis and characterisation of viral genome. Lancet 2013; 381:1916–25. 10.1016/S0140-6736(13)60903-4 23623390 PMC7134567

[9] Subbarao K , Klimov A, Katz J et al. Characterization of an avian influenza A (H5N1) virus isolated from a child with a fatal respiratory illness. Science 1998; 279: 393–6. 10.1126/science.279.5349.393 9430591

[10] Tran TH , Nguyen TL, Nguyen TD et al. Avian influenza A (H5N1) in 10 patients in Vietnam. N Engl J Med 2004; 350: 1179–88. 10.1056/NEJMoa040419 14985470

[11] Shi J , Deng G, Ma S et al. Rapid evolution of H7N9 highly pathogenic viruses that emerged in China in 2017. Cell Host Microbe 2018; 24: 558–68. 10.1016/j.chom.2018.08.006 30269969 PMC6310233

[12] World Health Organization/Global Influenza Programme . Monthly Risk Assessment Summary. https://www.who.int/influenza/human_animal_interface/Influenza_Summary_IRA_HA_interface_08_05_2020.pdf?ua=1 (5 August 2020, date last accessed).

[13] Wang Z , Zhang A, Wan Y et al. Early hypercytokinemia is associated with interferon-induced transmembrane protein-3 dysfunction and predictive of fatal H7N9 infection. Proc Natl Acad Sci USA 2014; 111: 769–74. 10.1073/pnas.1321748111 24367104 PMC3896201

[14] Zhang Q , Shi J, Deng G et al. H7N9 influenza viruses are transmissible in ferrets by respiratory droplet. Science 2013; 341: 410–4. 10.1126/science.1240532 23868922

[15] Galluzzi L , Vitale I, Aaronson SA et al. Molecular mechanisms of cell death: recommendations of the Nomenclature Committee on Cell Death 2018. Cell Death Differ 2018; 25: 486–541. 10.1038/s41418-017-0012-4 29362479 PMC5864239

[16] Shi J , Gao W, Shao F. Pyroptosis: gasdermin-mediated programmed necrotic cell death. Trends Biochem Sci 2017; 42: 245–54. 10.1016/j.tibs.2016.10.004 27932073

[17] Jorgensen I , Lopez JP, Laufer SA et al. IL-1beta, IL-18, and eicosanoids promote neutrophil recruitment to pore-induced intracellular traps following pyroptosis. Eur J Immunol 2016; 46: 2761–6. 10.1002/eji.201646647 27682622 PMC5138142

[18] Shi J , Zhao Y, Wang K et al. Cleavage of GSDMD by inflammatory caspases determines pyroptotic cell death. Nature 2015; 526: 660–5. 10.1038/nature15514 26375003

[19] Kayagaki N , Stowe IB, Lee BL et al. Caspase-11 cleaves gasdermin D for non-canonical inflammasome signalling. Nature 2015; 526: 666–71. 10.1038/nature15541 26375259

[20] Ding J , Wang K, Liu W et al. Pore-forming activity and structural autoinhibition of the gasdermin family. Nature 2016; 535: 111–6. 10.1038/nature18590 27281216

[21] Liu X , Zhang Z, Ruan J et al. Inflammasome-activated gasdermin D causes pyroptosis by forming membrane pores. Nature 2016; 535: 153–8. 10.1038/nature18629 27383986 PMC5539988

[22] Wang Y , Gao W, Shi X et al. Chemotherapy drugs induce pyroptosis through caspase-3 cleavage of a gasdermin. Nature 2017; 547: 99–103. 10.1038/nature22393 28459430

[23] Rogers C , Fernandes-Alnemri T, Mayes L et al. Cleavage of DFNA5 by caspase-3 during apoptosis mediates progression to secondary necrotic/pyroptotic cell death. Nat Commun 2017; 8: 14128. 10.1038/ncomms14128 28045099 PMC5216131

[24] Broz P , Pelegrin P, Shao F. The gasdermins, a protein family executing cell death and inflammation. Nat Rev Immunol 2020; 20: 143–57. 10.1038/s41577-019-0228-2 31690840

[25] Kovacs SB , Miao EA. Gasdermins: effectors of pyroptosis. Trends Cell Biol 2017; 27: 673–84. 10.1016/j.tcb.2017.05.005 28619472 PMC5565696

[26] Bergsbaken T , Fink SL, Cookson BT. Pyroptosis: host cell death and inflammation. Nat Rev Microbiol 2009; 7: 99–109. 10.1038/nrmicro2070 19148178 PMC2910423

[27] Rayamajhi M , Zhang Y, Miao EA. Detection of pyroptosis by measuring released lactate dehydrogenase activity. Methods Mol Biol 2013; 1040: 85–90. 10.1007/978-1-62703-523-1_7 23852598 PMC3756820

[28] Shi J , Deng G, Kong H et al. H7N9 virulent mutants detected in chickens in China pose an increased threat to humans. Cell Res 2017; 27: 1409–21. 10.1038/cr.2017.129 29151586 PMC5717404

[29] Imai M , Watanabe T, Kiso M et al. A highly pathogenic avian H7N9 influenza virus isolated from a human is lethal in some ferrets infected via respiratory droplets. Cell Host Microbe 2017; 22: 615–26. 10.1016/j.chom.2017.09.008 29056430 PMC5721358

[30] Wang Q , Wang Y, Ding J et al. A bioorthogonal system reveals antitumour immune function of pyroptosis. Nature 2020; 579: 421–6. 10.1038/s41586-020-2079-1 32188939

[31] Zhang Z , Zhang Y, Xia S et al. Gasdermin E suppresses tumour growth by activating anti-tumour immunity. Nature 2020; 579: 415–20. 10.1038/s41586-020-2071-9 32188940 PMC7123794

[32] Zhou Z , He H, Wang K et al. Granzyme A from cytotoxic lymphocytes cleaves GSDMB to trigger pyroptosis in target cells. Science 2020; 368: eaaz7548. 10.1126/science.aaz7548 32299851

[33] Kong H , Ma S, Wang J et al. Identification of key amino acids in the PB2 and M1 proteins of H7N9 influenza virus that affect its transmission in guinea pigs. J Virol 2019; 94: e01180–19. 10.1128/JVI.01180-19 31597771 PMC6912098

[34] Chen H , Li Y, Li Z et al. Properties and dissemination of H5N1 viruses isolated during an influenza outbreak in migratory waterfowl in western China. J Virol 2006; 80: 5976–83. 10.1128/JVI.00110-06 16731936 PMC1472608

[35] Sun Y , Li C, Shu Y et al. Inhibition of autophagy ameliorates acute lung injury caused by avian influenza A H5N1 infection. Sci Signal 2012; 5: ra16. 10.1126/scisignal.2001931 22355189

[36] Imai Y , Kuba K, Rao S et al. Angiotensin-converting enzyme 2 protects from severe acute lung failure. Nature 2005; 436: 112–6. 10.1038/nature03712 16001071 PMC7094998

[37] Teijaro JR , Walsh KB, Cahalan S et al. Endothelial cells are central orchestrators of cytokine amplification during influenza virus infection. Cell 2011; 146: 980–91. 10.1016/j.cell.2011.08.015 21925319 PMC3176439

