# Supplementary material for: H7N9 virus infection triggers lethal cytokine storm by activating gasdermin E-mediated pyroptosis of lung alveolar epithelial cells
Source: Natl Sci Rev. 2021 Jul 30;9(1):nwab137. doi: 10.1093/nsr/nwab137 (PMC8788236; doi:10.1093/nsr/nwab137)
Supplement: nwab137_Supplemental_Files [file nwab137_supplemental_files.zip › Supplementary_data_WanManuscript-R1.docx]

**Supplementary information**

Supplementary Data Figs. 1 to 8

**Supplementary Figure S1. Pulmonary chemokines and cytokines of mice infected with different H7N9 viruses.** Groups of six B6 mice were inoculated with 10^5.5^EID_50_ of the indicated virus or PBS as a control, and their lung lavage was collected on day 6 p.i. for quantification of chemokines and cytokines. Statistical analysis was performed by using the Student’s t-test. a, *p* < 0.01 compared with the values of AH/1-inoculated mice; b, *p* < 0.05 compared with the values of PG/S1421-inoculated mice.

**Supplementary Figure S2. Examination of gasdermin E (GSDME) expression in normal human lung.** GSDME expression in human lung alveolar epithelial cells was detected by using an immunohistochemical (IHC) assay with a mouse anti-DFNA5/GSDME monoclonal antibody as the primary antibody and horse anti-mouse IgG, HRP-linked antibody as the secondary antibody. Scale bar = 100 μm.**Supplementary Figure S3. Morphological changes of different cell types after AH/1 infection.** The indicated cells (5×10^5^/well) were seeded into 6-well plates, and 24 h later the cells were infected with virus at an MOI of 5. The mouse lung alveolar epithelial cells Mle12 (**A**) undergo pyroptosis upon AH/1 infection, and different macrophages (**B** to **D**) undergo apoptosis upon AH/1 infection. Static bright field cell images were obtained at 48 h post-infection by using the Zeiss primovert microscope and were processed by using zen blue software.

**Supplementary Figure S4. Replication of AH/1 in different cell types**. The indicated cells (2×10^5^/well) were seeded into 12-well plates, and 24 h later the cells were infected with AH/1 virus at an MOI of 0.5; their supernatants were harvested at the indicated timepoints for viral titer detection in chicken eggs. *, *p* < 0.05 compared with the values for the A549-vector cells. **, *p* < 0.05 compared with the values for the Raw-vector cells.

**Supplementary Figure S5. Pulmonary chemokines and cytokines of wild-type B6 and Gsdme-/- mice infected with AH/1 virus.** Groups of five B6 or *Gsdme-/-* mice were inoculated with 10^5.5^EID_50_ of the AH/1 virus, and their lung lavage was collected on day 6 p.i. for quantification of chemokines and cytokines. Data are means ± standard deviations. Statistical analysis was performed by using the Student’s t-test. a, *p* < 0.01 compared with the values for wild-type mice.

**Supplementary Figure S6. Morphological changes of and LDH release by A549 cells after infection with different of H7N9 viruses.** A549 cells (5×10^5^/well) were seeded into 6-well plates, and 24 h later the cells were infected with PG/S1421 or AH/1 at the indicated dose. (**A**) Static bright field cell images were obtained at 48 h post-infection by using the Zeiss primovert microscope and were processed by using zen blue software. (**B**) LDH release was measured at 48 h post infection. Data are means ± standard deviations. Statistical analysis was performed by using the Student’s t-test.

**Supplementary Figure S7. Morphological changes of and LDH release by A549 cells and A549-GSDME/- cells after infection with H5N1 virus BHG/3.** (**A**) GSDME expression in A549 cells and A549-GSDME/- cells. (**B**) A549 cells (5×10^5^/well) were seeded into 6-well plates, and 24 h later the cells were infected with BHG/3 virus at an MOI of 5. Static bright field cell images were obtained at 48 h post-infection by using the Zeiss primovert microscope and were processed by using zen blue software. (**C**) LDH release was measured at the indicated timepoints post-infection.

**Supplementary Figure S8. Replication and virulence of H5N1 virus BHG/3 in wild-type B6 and *Gsdme*^-/-^ mice.** Groups of 22 wild-type B6 mice and *Gsdme* knockout (*Gsdme*^-/-^) mice were intranasally inoculated with 10 MLD_50_ of BHG/3 virus. Five mice in each group were euthanized on days 3 and 6 p.i., respectively, and their organs were collected for virus titration in eggs (**A**). The remaining 10 mice in each group were used to evaluate lethality and their body weight change (**B**) and death (**C**) were observed for two weeks.

**Online Methods**

**Facility, Ethics, and Biosafety statement**

All experiments with infectious H5N1 and H7N9 viruses were performed in the enhanced biosafety level 3 (P3+) and animal P3+ facilities in the Harbin Veterinary Research Institute (HVRI) of the Chinese Academy of Agricultural Sciences (CAAS), which is approved for such use by the Ministry of Agriculture and Rural Affairs of China. The animal studies were carried out in strict accordance with the recommendations in the Guide for the Care and Use of Laboratory Animals of the Ministry of Science and Technology of the People’s Republic of China. The protocols were approved by the Committee on the Ethics of Animal Experiments of the HVRI of CAAS (Approval number 2017-08-08HS).

All activities inside the P3+ labs were performed by authorized personnel that have received appropriate training. The researchers wore powered air purifying respirators that filtered the air, and disposable coveralls when they worked inside the facility. The researchers were disinfected before leaving the room and showered on exiting the facility.

**Cells, antibodies, and reagents**

The HEK293T, Mle12, Raw264.7, and THP-1 cell lines were obtained from the American Type Culture Collection. The human primary alveolar epithelial cells were from ScienCell Research Laboratories (California, USA). The human primary type Ⅱ alveolar epithelial cells were from AcceGen (New Jersey, USA). The human CD14+ monocytes were from AllCells (California, USA). The murine primary macrophages (mø) were prepared from Thioglycolate (Merk, 1.08191.0500)-elicited peritoneal macrophages. Human normal lung tissues were obtained from Shanghai Biochip (Shanghai, China).

Rabbit anti-NP polyclonal and mouse anti-NP monoclonal antibodies were generated in our laboratory by using conventional methods. Rabbit anti-DFNA5/GSDME monoclonal antibody [EPR19859] [catalog number (CN): ab215191], rabbit anti-TTF1 monoclonal antibody [EP1584Y] [CN: ab76310] were from Abcam (Cambridge, UK). Mouse anti-DFNA5/GSDME monoclonal antibody [G9] [CN: sc-393162] used in the IHC assay was from Santa Cruz Biotechnology (Texas, USA). Rabbit anti-caspase-3 monoclonal antibody (CN: 14220), rabbit anti-cleaved caspase-3 monoclonal antibody (CN: 9664), rabbit anti-F4/80 monoclonal antibody (CN: 70076), rabbit anti-β-actin monoclonal antibody (CN: 4970), and goat anti-rabbit IgG, HRP-linked antibody (CN: 7074) were from Cell Signaling Technology (Beverly, MA). Rabbit anti-aquaporin 5 polyclonal antibody [CN: BA2205] was from Boster (Wuhan, China). Anti-rabbit IgG (whole molecule)-HRP (CN: A-9169) was from Sigma-Aldrich. Goat anti-mouse IgG (H+L)-cy3 (CN: A0521) was from Beyotime Biotechnology (Shanghai, China). Goat anti-rabbit IgG (H+L)-Fluorescein (CN: F1-1000) was from Vector Laboratories (California, USA). Rat anti-mouse CD16/32 monoclonal antibody (CN: 101330), rat anti-mouse/human APC-conjugated CD11b (M1/70) monoclonal antibody (CN: 101212), rat anti-mouse PE-conjugated F4/80 monoclonal antibody (CN: 123110), rat anti-mouse Alexa Fluor488-conjugated CD45 monoclonal antibody (CN: 103121), and rat anti-mouse APC-conjugated Ly6G monoclonal antibody (CN: 127613) were from Biolegend (San Diego, CA).

Annexin V-APC/PI apoptosis kit (CN: 88-8007-72) was from eBioscience (San Diego, CA). Cyto Tox 96 Non-Radioactive Cytotoxicity kit (CN: G1780) was from Promega (Madison, WI).

**Viruses**

The isolation and characterization of the viruses A/Anhui/1/2013 (H7N9) (AH/1), A/pigeon/Shanghai/S1421/2013 (H7N9) (PG/S1421), CK/SD008-PB2/627K (H7N9), and A/bar-headed goose/Qinghai/3/2005 (H5N1) (BHG/3) were described previously [1,14,28,34]. Viral stocks of these strains were propagated and titrated in 10-day-old specific-pathogen-free embryonated chicken eggs.

**Mouse studies**

Wild-type C57BL/6 (B6) mice were purchased from Vital River Laboratory Animal Technology Co.; the *Gsdme-/-* mice were generated by co-microinjection of in vitro-translated Cas9 mRNA and gRNAs into B6 zygotes as reported previously [22]. *Gsdme-/-* mice were hybridized with wild-type C57BL/6 (B6) mice, and littermate wild-type and *Gsdme-/-* mice were used. Randomization and blinding were not used for the allocation of animals to experimental groups. The number of animals used in each study was determined by following the “minimum quantity-principle” in our protocol. Most of the animal studies were performed once, unless otherwise noted, and the biological replicates are indicated in detail below.

To compare the viral replication and virulence of AH/1 and PG/S1421 in wild-type B6 mice, groups of 32 6-week-old female B6 mice were inoculated with 10^5.5^ 50% egg infectious dose (EID_50_) of AH/1 or PG/S1421, and 16 similar-aged B6 mice were inoculated with PBS as a control. Five mice in each virus-inoculated group were euthanized on days 3 and 6 post-infection (p.i.), respectively, and their nasal turbinate and lungs were collected for virus titration. Six mice in each virus-inoculated group were observed for body weight change and death for two weeks. Lung lavage samples from six mice in each virus-infected group and the PBS control group were collected for analysis of chemokines and cytokines on day 6 p.i.. Lungs from 10 mice in each virus-infected group and the PBS control group were collected on day 6 p.i. for assessing pulmonary edema (n = 5) and quantification of infiltration of immune cells by flow cytometry (n = 5).

To investigate caspase-3 activation and GSDME cleavage in the lungs of mice inoculated with H7N9 viruses, groups of 12 6-week-old B6 mice were inoculated with 10^5.5^ EID_50_ of AH/1 or PG/S1421 virus, three mice in each group were euthanized on day 6 p.i. and their lungs were collected for pathologic and immunohistochemical (IHC) studies to detect the viral antigen and activated caspase-3. Three mice in each virus-inoculated group were euthanized on days 3, 5, and 7 p.i., respectively, and their lungs were collected for the detection of caspase-3 and GSDME by use of SDS-PAGE and Western blotting.

To evaluate viral replication and virulence in different types of mice, groups of wild-type B6 and *Gsdme-/-* mice were intranasally inoculated with 10 MLD_50_ of AH/1 (n = 28), CK/SD008-PB2/627K (n = 20), or BHG/3 (n = 22). Five mice from each group were euthanized on days 3 and 6 p.i., respectively, and their organs were collected for virus titration in eggs. Three mice in each of the AH/1-inoculated groups were euthanized on day 6 p.i., and their lungs were collected for pathologic study. Five mice in each of the AH/1-inoculated groups were euthanized on day 6 p.i., and their lung lavages were collected for analysis of different cytokines. The remaining mice from each group were monitored for virus lethality for two weeks; body weight changes and death of the mice were daily recorded. The lethality tests of AH/1 and CK/SD008-PB2/627K in wild-type and *Gsdme-/-* mice were repeated once.

**Mouse pulmonary chemokine and cytokine quantification**

The lungs of each test mouse were collected and placed on 6-cm cell culture dishes on ice and washed repeatedly with 1 ml of cold PBS using a syringe. Virus in the lung lavage was inactivated by using an Ultraviolet Crosslinker under 650.0 mj/cm^2^ for 20 minutes and the virus inactivation was confirmed by egg propagation. The chemokine and cytokine levels in the lung lavage were determined by using the Multiplex suspension array system (Thermo fisher Scientific).

**Lung wet/dry weight ratio**

To assess pulmonary edema, the lungs of each virus-inoculated or control mouse were collected and weighed to obtain the wet mass. The lungs were then heated to 65°C in a gravity convection oven for 24 h and weighed to obtain the dry mass. The wet/dry weight ratio was calculated by dividing the dry mass by the wet mass [36].

**Quantification of macrophages and neutrophils by using flow cytometry**

The lungs of each virus-inoculated or control mouse were collected and chopped into 1–2 mm^2^ pieces and then suspended in 5 ml of lysis buffer [2 mg/ml Type IV collagenase A (Life technology), 10 units/ml Deoxyribonuclease I (Life technology)] for 1.5 h at 37°C. The dissociated single cells were filtered through a 70-μm ﬁlter (BD Bioscience) and the red blood cells were lysed by using red blood cell lysis buffer (Solarbio). Total lung cells for each mouse were counted and their Fc receptors were blocked by using 25 mg/ml anti-mouse CD16/32 antibody [37]. A total of 10^6^ cells from each mouse were then stained with Alexa Fluor488-conjugated CD45, APC-conjugated CD11b, PE-conjugated F4/80, and APC-conjugated Ly6G antibodies according to manufacturers’ instructions. The macrophages (APC-conjugated CD11b and PE-conjugated F4/80 double-positive cells) and neutrophils (APC-conjugated Ly6G positive cells) of the gated Alexa Fluor488-conjugated CD45 population of lung cells were identified by flow cytometry using Accuri C6 plus (BD). The data were then analyzed with FlowJo software.

**Histologic studies**

The lungs of mice were fixed in 10% neutral-buffered formalin, embedded in paraffin, and cut into 4-µm sections. The sections were stained with hematoxylin-eosin (H&E) or used in immunohistochemical (IHC) assays. The sections used for immunohistochemistry were dewaxed in xylene and hydrated through a series of descending concentrations of alcohol to water. For viral antigen retrieval, sections were immersed in citric acid/sodium citrate solution at 121 °C for 15 minutes. After cooling, the sections were treated with 3% hydrogen peroxide for 30 minutes to remove endogenous peroxidase activity and blocked with 8% skim milk to reduce nonspecific binding. After three 5-minute washes in TBS, the sections were incubated with rabbit antiserum against avian influenza nucleoprotein or rabbit anti-cleaved caspase-3 antibody in 8% skim milk at 4 °C overnight. The sections were then washed again with TBS and incubated with goat anti-rabbit IgG (whole molecule)-HRP at room temperature for 60 minutes. The immunostaining was visualized with DAB and counterstained with hematoxylin.

**Dual-staining assay**

Double-labeling immunofluorescence histochemistry was performed by using the mouse anti-avian influenza virus nucleoprotein (NP, 1:50) and either the rabbit anti-TTF1 (1:200, Abcam), rabbit anti-Aquaporin 5 (1:100, Boster), rabbit anti-F4/80 (1:100 Cell Signaling) or rabbit anti-cleaved caspase-3 antibody (1:100, Cell Signaling). Lung sections of mouse infected with AH/1 virus were first incubated with the mouse anti-avian influenza virus nucleoprotein antibody as the primary antibody and then visualized with goat anti-mouse IgG (H+L)-cy3. The sections were then washed with TBS and incubated with either the rabbit anti-TTF1 antibody, rabbit anti-Aquaporin 5 antibody, rabbit anti-F4/80 antibody, or rabbit anti-cleaved caspase-3 antibody. The cell marker (TTF1, Aquaporin 5 and F4/80) or cleaved caspase 3 was visualized by using goat anti-rabbit IgG (H+L)-fluorescein antibody. Sections were mounted with Vectashield (Vector Laboratories) and observed by using the Zeiss LSM700 confocal laser scanning microscope.

**Detection of activated caspase-3 and cleaved GASDME in the lungs of mice by Western blotting**

Mice were euthanized on different days after virus inoculation, and total mouse lung protein was extracted using a total animal tissue extract kit (Invent, SD001) and its concentration was measured by using a bicinchoninic acid assay (Pierce, 23235). Equal amounts of protein from each mouse were loaded for SDS-PAGE. The proteins on the gel were then transferred onto polyvinylidene fluoride membranes (Bio-rad), blocked with 5% skim milk in Tris-buffered saline (pH 7.4) containing 0.1% Tween-20, and then incubated overnight at 4°C with rabbit anti-DFNA5/GSDME monoclonal antibody, rabbit anti-caspase-3 monoclonal antibody, rabbit anti-cleaved caspase-3 monoclonal antibody, and rabbit anti-β-actin monoclonal antibody, respectively. After incubation with HRP-coupled secondary antibody, the blots were visualized by using the electrochemiluminescence method (Pierce) and the Fluor Chem E imaging system (ProteinSimple).

**Generation of the GSDME-deficient A549 cell line (A549-GSDME/-)**

The siRNA (5’-CCAUUGCCUACGGUGUCAUTT-3’) targeting human GSDME and scrambled siRNA (5’-UUCUCCGAACGUGUCACGUTT-3’) were respectively cloned into the lentiviral shRNA expression vector pLKO.1-GFP (Addgene Inc, Watertown, MA) and the resultant plasmids were designated pLKO.1-GSDME and pLKO.1-vector, respectively. To construct the GSDME-deficient A549 cell line (A549-GSDME/-) and the A549 control cell line (A549-vector), recombinant plasmids pLKO.1-GSDME or pLKO.1-vector were co-transfected into HEK293T cells with the packing plasmids pMD2G and pSPAX2 (Addgene Inc, Watertown, MA) at a ratio of 4:3:2 by using JetPRIME reagent (Polyplus Transfection) and following the manufacturer’s instructions. The supernatant was collected 48 h post-transfection and used to infect A549 cells. After being cultured for 48 h, the GFP-positive A549 cells were sorted into single clones and seeded in 96-well plates by use of flow cytometry using the Beckman Coulter MoFlo XDP cell sorter. GSDME expression in the cloned cells was verified by western blotting.

**Generation of a stable GSDME-overexpressing Raw264.7 cell line (Raw-GSDME/+)**

The cDNA encoding murine GSDME amplified from Mle12 cells was cloned into the lentiviral expression vector pCDF1-GFP (System Bioscience), and the recombinant plasmid was designated pCDF1-GFP-GSDME. To construct the GSDME-overexpressing Raw264.7 cell line (Raw-GSDME/+) and negative control cell line (Raw-vector), HEK293T cells were transfected with pCDF1-GFP-GSDME or empty pCDF1-GFP plasmid, together with the packing plasmids pMD2G and pSPAX2 at a ratio of 4:3:2 by using Jet PRIME reagent (Polyplus Transfection). Lentiviruses were harvested 48 h post-transfection and used to infect Raw264.7 cells. GFP-positive infected cells were sorted into single clones in 96-well plates by use of flow cytometry using the Beckman Coulter MoFlo XDP cell sorter. GSDME expression in the cloned cells was verified by western blotting.

**Detection of cellular GSDME by Western blotting**

To evaluate the cellular GSDME levels of different cells, total protein of cultured cells was extracted with lysis buffers supplemented with protease inhibitor cocktail (Cell Signaling Technology) by following the manufacturer’s instructions. Protein concentrations of the extracts were measured by using the bicinchoninic acid assay (Pierce). Equal amounts of total protein were loaded per lane for SDS-PAGE. The proteins on the gel were then transferred onto polyvinylidene fluoride membranes (Bio-rad) and blocked with 5% skim milk in Tris-buffered saline (pH 7.4) containing 0.1% Tween-20. The blots were incubated overnight at 4°C with rabbit anti-DFNA5/GSDME monoclonal antibody and rabbit anti-β-actin monoclonal antibody. After incubation with HRP-coupled secondary antibody, the blots were visualized by using the ECL method (Pierce) and the Fluor Chem E imaging system (ProteinSimple).

**Detection of activated (cleaved) caspase-3 and cleaved GASDME in different cell types by Western blotting**

To evaluate the activated (cleaved) caspase-3 and cleaved GASDME of different cells after AH/1 virus infection, HPAE, HPAE Ⅱ, A549, and Mle12 cells were infected with AH/1 virus at an MOI of 5. Supernatants and cells were harvested at the indicated timepoints for total protein extraction with lysis buffers supplemented with protease inhibitor cocktail (Cell Signaling Technology). Protein concentrations of the extracts were measured by using the bicinchoninic acid assay (Pierce). Equal amounts of total protein were loaded per lane for SDS-PAGE. The proteins on the gel were then transferred onto polyvinylidene fluoride membranes (Bio-rad) and blocked with 5% skim milk in Tris-buffered saline (pH 7.4) containing 0.1% Tween-20, and then were incubated overnight at 4°C with rabbit anti-DFNA5/GSDME monoclonal antibody, rabbit anti-caspase-3 monoclonal antibody, rabbit anti-cleaved caspase-3 monoclonal antibody, and rabbit anti-β-actin monoclonal antibody, respectively. After incubation with HRP-coupled secondary antibody, the blots were visualized by using the electrochemiluminescence method (Pierce) and the Fluor Chem E imaging system (ProteinSimple).

**Observation of morphological changes in influenza virus-infected cells**

To observe morphologic changes, cells (5×10^5^/well) were seeded into 6-well plates, and 24 h later were infected with AH/1 virus at an MOI of 5. Static bright field cell images were obtained using the Zeiss primovert microscope and processed using zen blue software. The time-lapse phase-contrast and fluorescent images of cells were taken at the indicated timepoints after viral infection and labelling with green membrane dye 3,3'-Dioctadecyloxacarbocyanine Perchlorate (DIO) (Thermo fisher Scientific) by using the PerkinElmer UltraVIEW spinning disk confocal microscope and were processed by using the Volocity software.

**Quantification of propidium iodide-stained pyroptotic cells**

Cells in 6-well plates were infected with virus at an MOI of 5 and 24 h later the cells were collected, washed twice, and stained with the Annexin V-APC/PI apoptosis kit according to manufacturer’s instructions (eBioscience). The stained cells were identified by means of flow cytometry using A60-UNIVERSAL minutes (apogee). The data were analyzed with FlowJo software.

**Lactate dehydrogenase (LDH) release test**

Cells were seeded into 48-well plates (2×10^4^ cells/well) and then infected with virus at an MOI of 5. The supernatant was harvested at the indicated timepoints to measure LDH release by using the Cyto Tox 96 Non-Radioactive Cytotoxicity kit according to the manufacturer’s instructions (Promega, Madison, WI).

**Replication of AH/1 in different cell types**

To evaluate H7N9 virus replication in cells with different GSDME levels, A549-vector, A549-GSDME/-, Raw-vector, and Raw-GSDME/+ cells (2×10^5^/well) were seeded into 12-well plates, and 24 h later the cells were infected with AH/1 virus at an MOI of 0.5. Cell supernatants were harvested at 12, 24, 36, and 48 h p.i. for viral titer detection in chicken eggs.

**Statistical analysis**

Data are presented as means ± standard deviations. Statistical analyses were performed using GraphPad Prism6 software. Statistical significance was calculated by using the Student’s two-tailed unpaired t-test.
