# Supplementary figures and images for: H7N9 virus infection triggers lethal cytokine storm by activating gasdermin E-mediated pyroptosis of lung alveolar epithelial cells
Source: Natl Sci Rev. 2021 Jul 30;9(1):nwab137. doi: 10.1093/nsr/nwab137 (PMC8788236; doi:10.1093/nsr/nwab137)

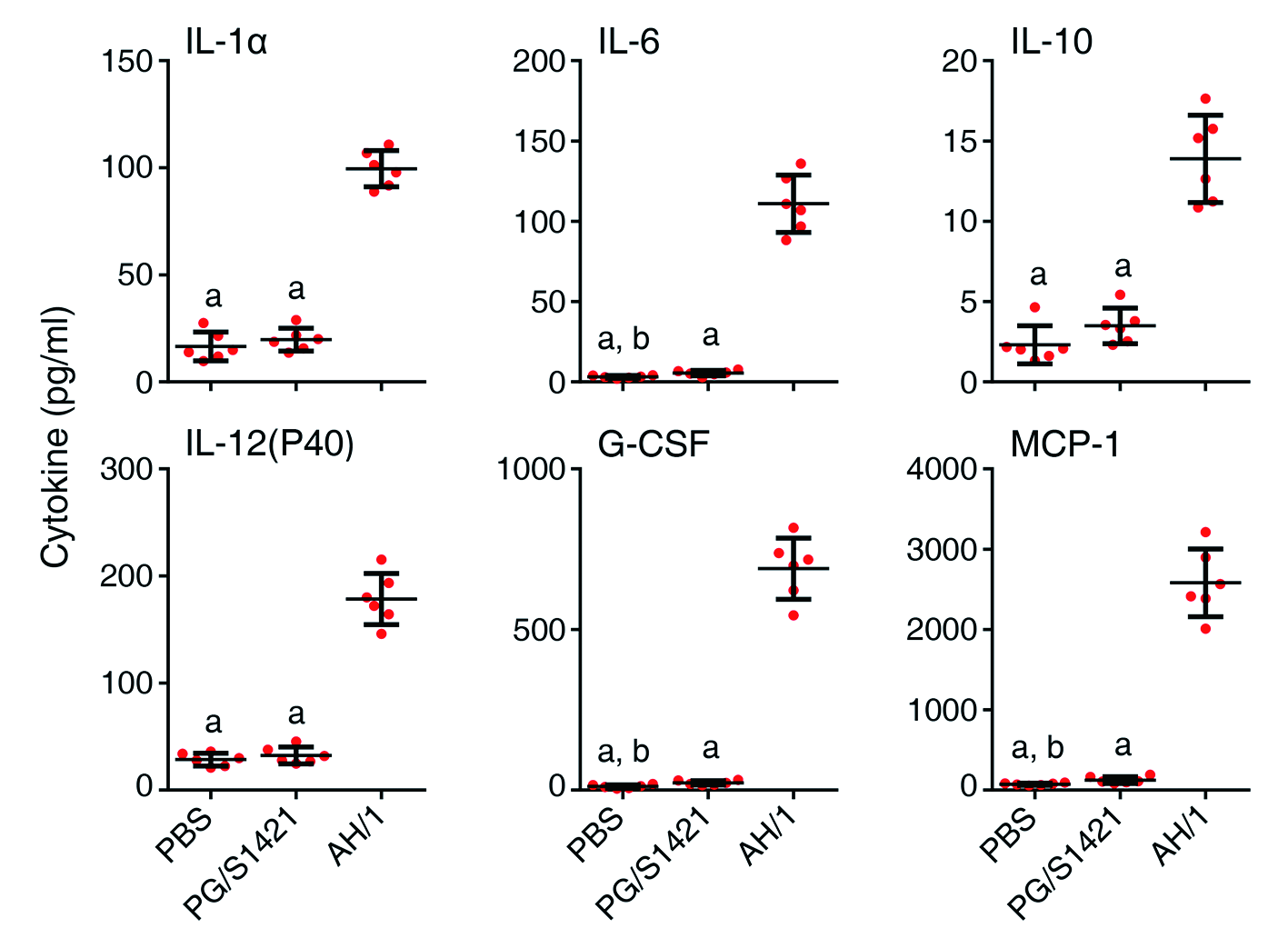

Supplement: nwab137_Supplemental_Files [file nwab137_supplemental_files.zip › NSR_MS-2021-811_Supplementary_Fig_S1.tif]

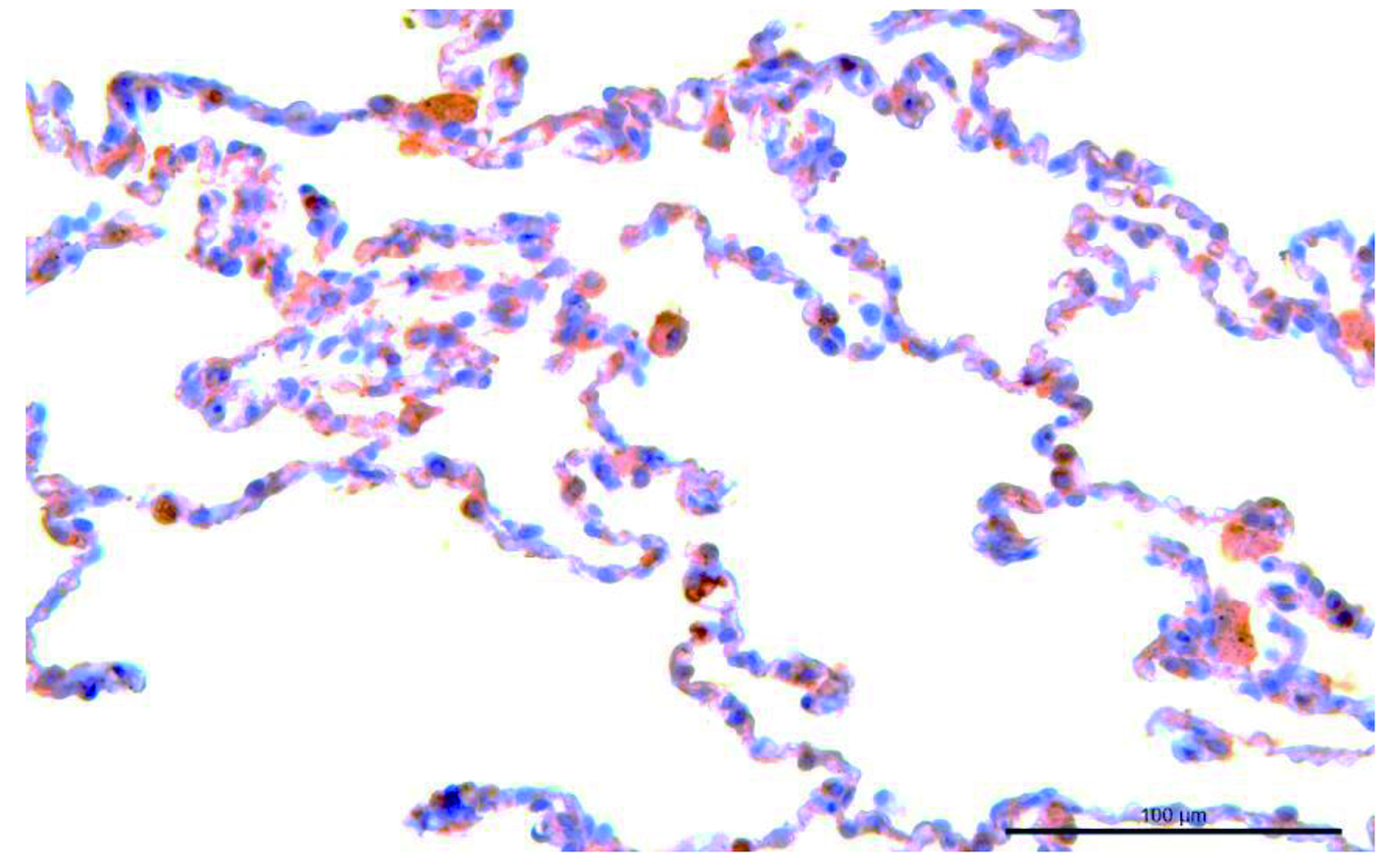

Supplement: nwab137_Supplemental_Files [file nwab137_supplemental_files.zip › NSR_MS-2021-811_Supplementary_Fig_S2.tif]

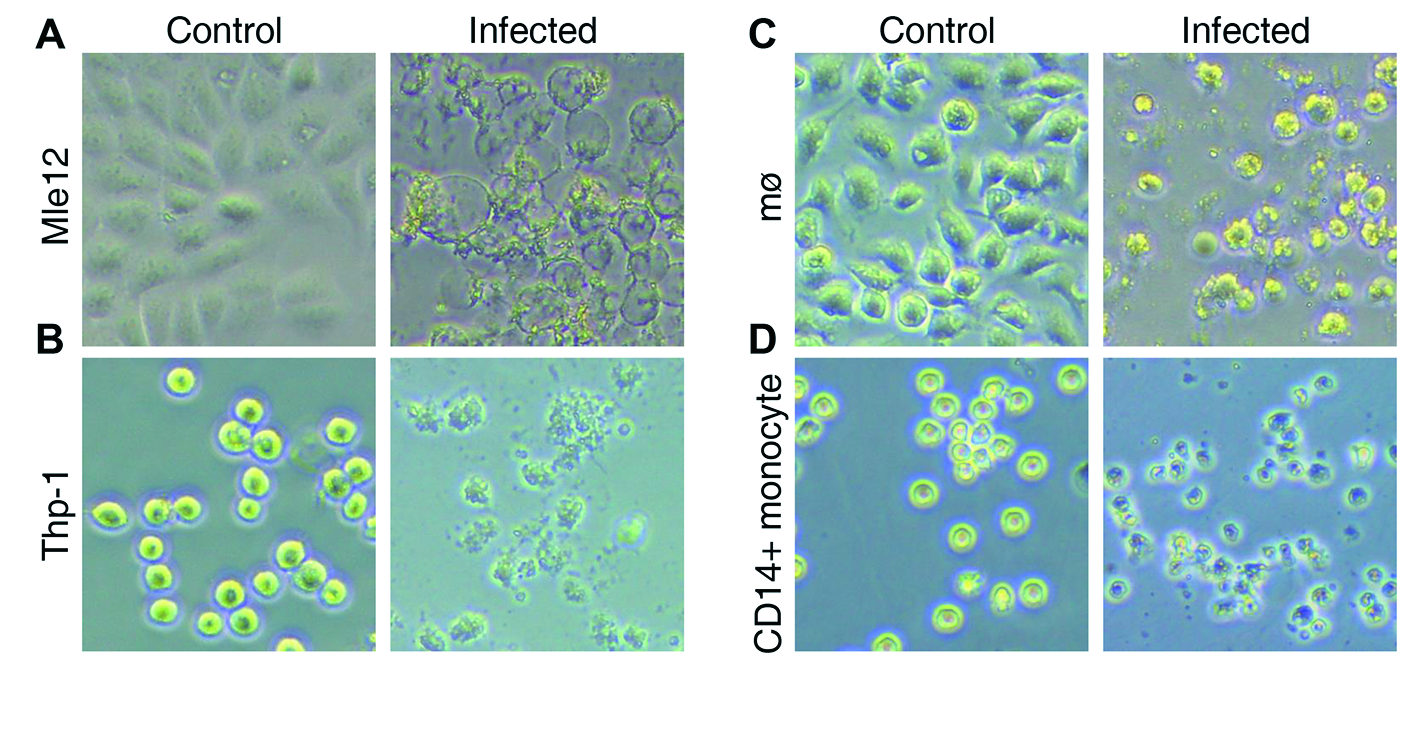

Supplement: nwab137_Supplemental_Files [file nwab137_supplemental_files.zip › NSR_MS-2021-811_Supplementary_Fig_S3.tif]

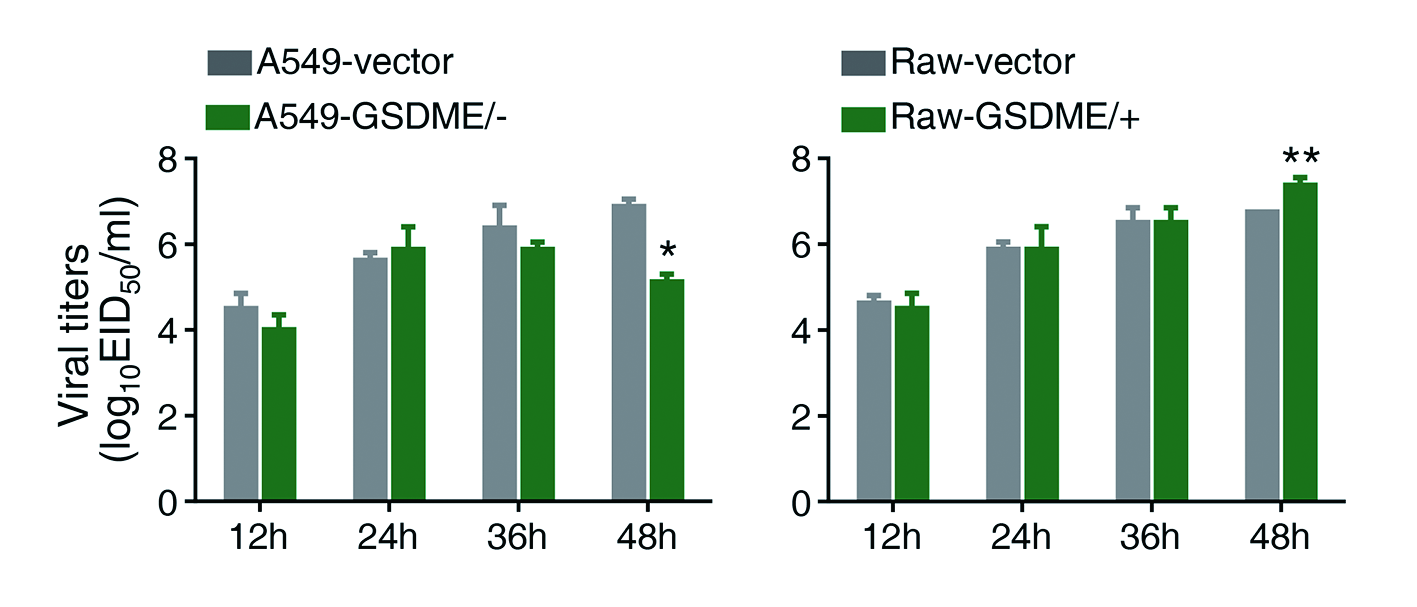

Supplement: nwab137_Supplemental_Files [file nwab137_supplemental_files.zip › NSR_MS-2021-811_Supplementary_Fig_S4.tif]

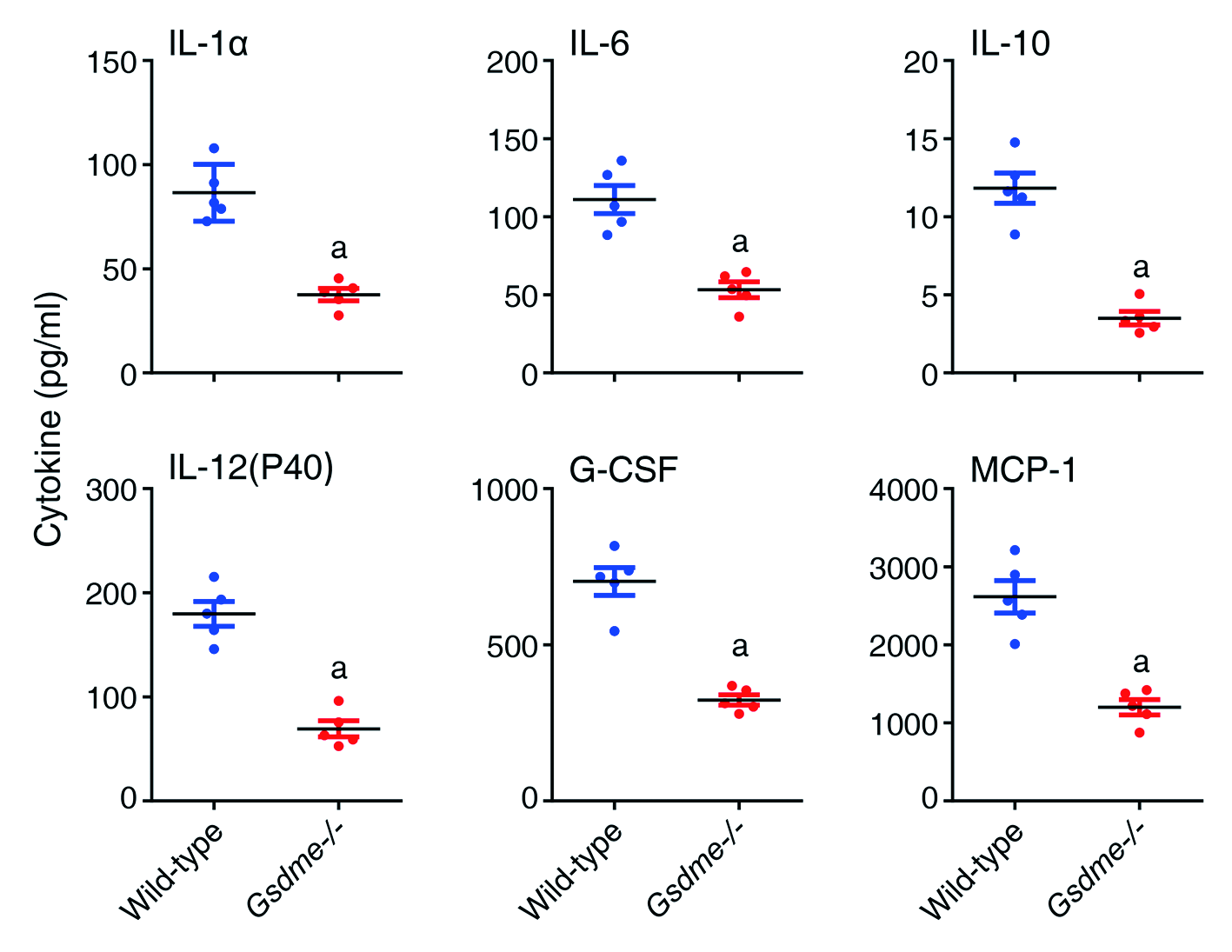

Supplement: nwab137_Supplemental_Files [file nwab137_supplemental_files.zip › NSR_MS-2021-811_Supplementary_Fig_S5.tif]

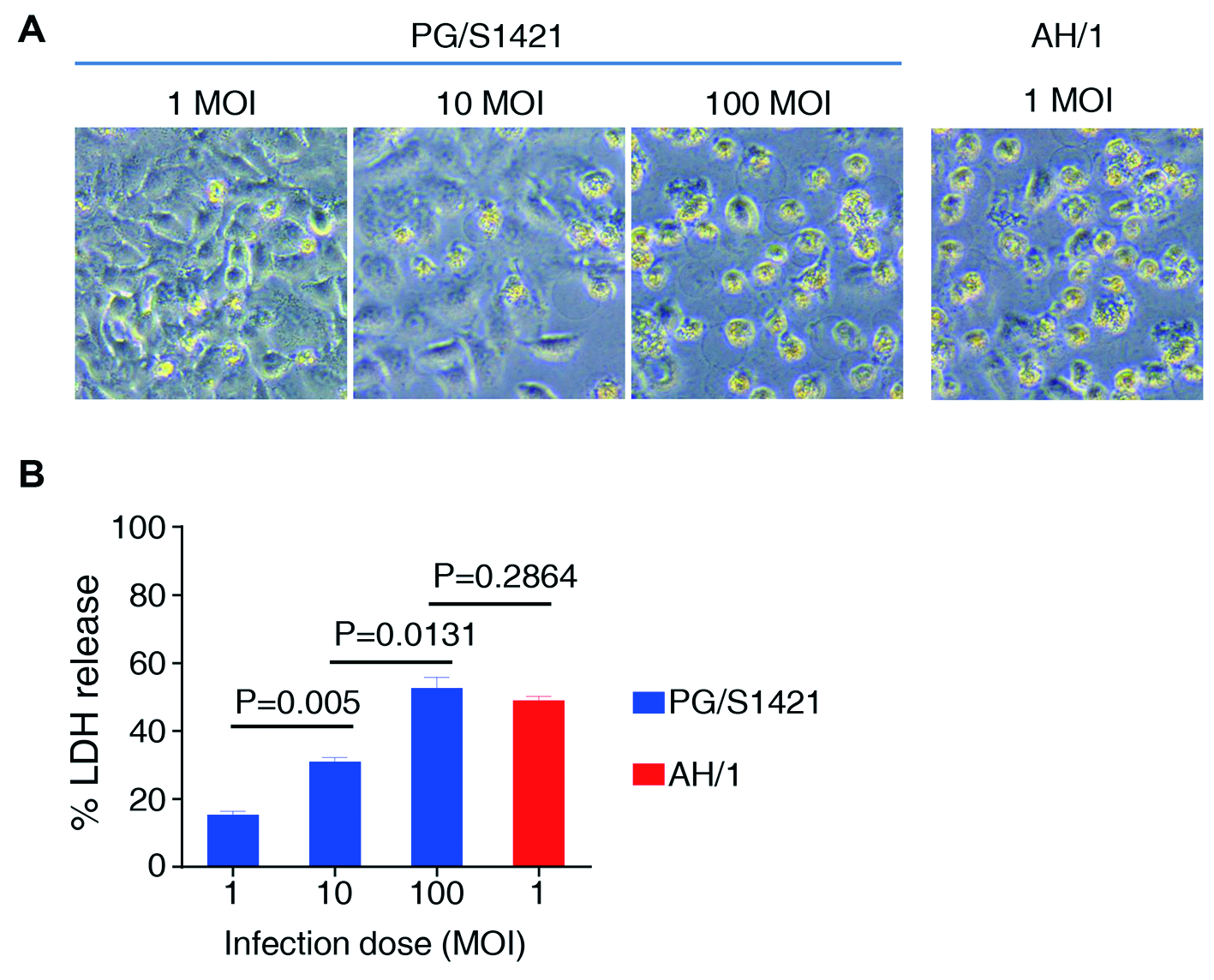

Supplement: nwab137_Supplemental_Files [file nwab137_supplemental_files.zip › NSR_MS-2021-811_Supplementary_Fig_S6.tif]

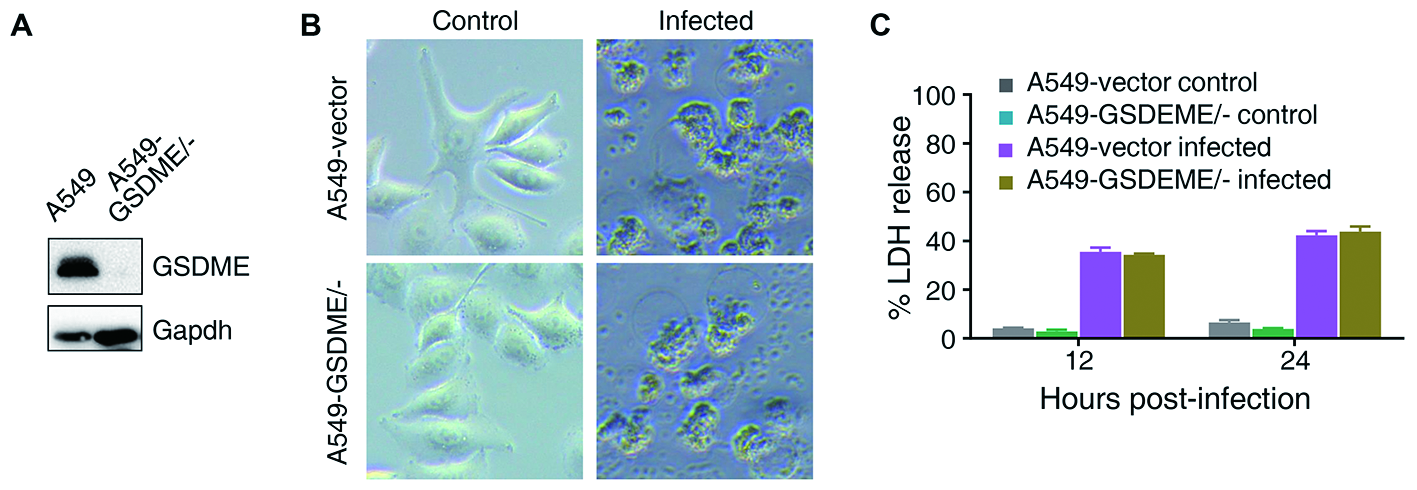

Supplement: nwab137_Supplemental_Files [file nwab137_supplemental_files.zip › NSR_MS-2021-811_Supplementary_Fig_S7.tif]

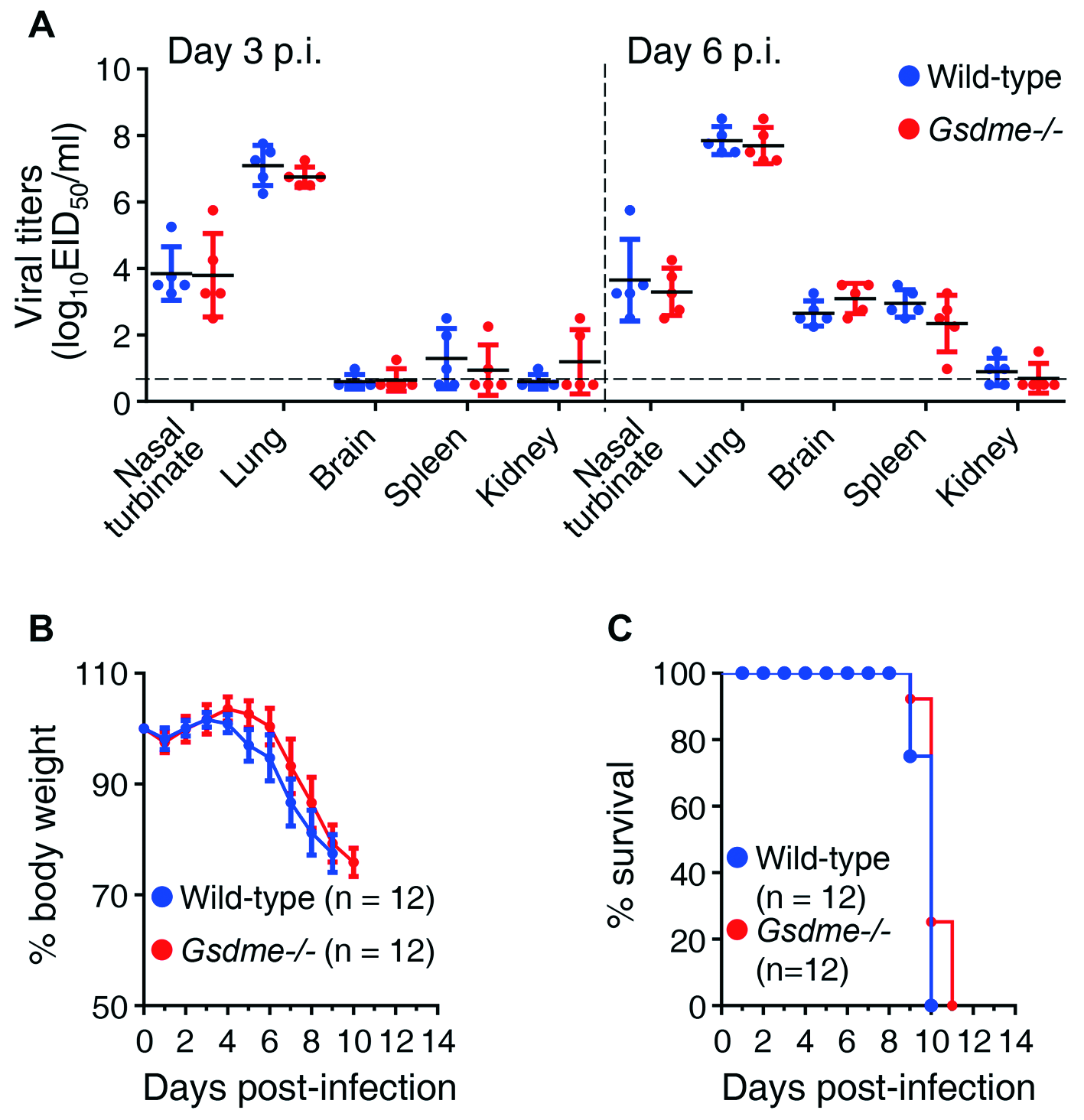

Supplement: nwab137_Supplemental_Files [file nwab137_supplemental_files.zip › NSR_MS-2021-811_Supplementary_Fig_S8.tif]
